# Modified Support Vector Machine for Detecting Stress Level Using EEG Signals

**DOI:** 10.1155/2020/8860841

**Published:** 2020-08-01

**Authors:** Richa Gupta, M. Afshar Alam, Parul Agarwal

**Affiliations:** Department of Computer Science and Engineering, School of Engineering and Technology, Jamia Hamdard, Delhi 110062, India

## Abstract

Stress is categorized as a condition of mental strain or pressure approaches because of upsetting or requesting conditions. There are various sources of stress initiation. Researchers consider human cerebrum as the primary wellspring of stress. To study how each individual encounters stress in different forms, researchers conduct surveys and monitor it. The paper presents the fusion of 5 algorithms to enhance the accuracy for detection of mental stress using EEG signals. The Whale Optimization Algorithm has been modified to select the optimal kernel in the SVM classifier for stress detection. An integrated set of algorithms (NLM, DCT, and MBPSO) has been used for preprocessing, feature extraction, and selection. The proposed algorithm has been tested on EEG signals collected from 14 subjects to identify the stress level. The proposed approach was validated using accuracy, sensitivity, specificity, and *F*1 score with values of 96.36%, 96.84%, 90.8%, and 97.96% and was found to be better than the existing ones. The algorithm may be useful to psychiatrists and health consultants for diagnosing the stress level.

## 1. Introduction

Mental stress has become a social problem of the 21^st^ century. It affects the functionality of the routine work and economy of an individual human and a nation as well. Stress can be classified as positive and negative. Positive stress alerts us and avoids the danger leading to performance enhancement while negative stress can cause mental and behavioral changes as one does not get relaxation in between various challenges. Enthusiastic stress emerges because of work pressure, complying with up time constraint, tests, and so on [1]. There are various sources of stress initiation [2]. Researchers consider human cerebrum as the primary wellspring of stress [3, 4]. To study the effect of stress on different individuals, surveys are conducted and even monitoring of individuals takes place [5]. Self-report surveys are one of the most regularly utilized techniques to gauge a person's degree of stress [6–8]. To distinguish human feelings and stress discovery, electroencephalogram (EEG) is a significant technique used in stress detection and has shown some significant results [9]. Electroencephalography (EEG) is a test to estimate and record the brain's electrical activity [10, 11]. The handling of EEG signals is the most basic part in the present investigations [12]. Various techniques have been developed either on the basis of a questionnaire or quantifying the changes in physiological signals to measure and study the stress level. Physiological signals are online real-time system and gives better accuracy in stress estimation. These can be classified into two types: (i) invasive type and (ii) noninvasive type. Invasive type methods such as local field potential (LFP) and electrocorticography (EcoG) provide high resolution in temporal and spatial axes and high specificity. However, they have limited coverage and may be not suitable to all situations. The noninvasive methods such as (i) functional magnetic resonance imaging (fMRI), (ii) electroencephalography (EEG), and (iii) magnetic encephalography (MEG) are presently used for assessment of stress effect on human health. EEG and MEG have higher resolution whereas fMRI has high spatial resolution but very low temporal resolution.

The proposed modified Whale Optimization Algorithm (WOA) selects the optimal kernel in the support vector machine (SVM) classifier for stress detection. Besides, the other main contributions of this paper are the following:

1. We integrated a certain set of algorithms (namely, NLM, DCT, and MBPSO) for preprocessing, feature extraction, and selection from noisy EEG.
2. Optimal set of features which depend on the frequency level are selected using MBPSO technique.
3. The Kernel of SVM is modified using WOA, since the optimal SVM machine learning classifier has been used for long term stress classification in which it attains the better performance.
4. Also, the proposed algorithm has been tested to identify the stress level among 14 subjects.
5. Further, the results of modified and existing algorithm have been compared to identify the efficiency of the 2 algorithms. Here, the authors want to mention that WOA has not been used in any neurological application and an attempt has been made by the authors to observe this algorithm effect in identifying stress through EEG signals.

This paper is divided into 5 sections.  Section 1 explains the stress behavior and its analysis in Introduction, Section 2 explores the different research work carried out in the related area, Section 3 explains the methodology proposed in the paper, and Section 4 explains and discusses the results obtained after implementing our methodology on our original work and presents comparison with modified version of our original implemented work.  Section 5 concludes the study of this research paper.

## 2. Literature Review

A lot of work has been carried out on detecting the stress. Some are discussed in this section.

In [13], authors have enhanced some statistical technique using Renyi's entropy to develop a technique for removing artifacts that get introduced during the EEG recordings.

Authors in [14] had stated the difficulty in removing artifacts present in biomedical signals. To overcome this difficulty, they had developed a method where they used discrete wavelet transform (DWT) and independent component analysis (ICA) together. They tested their method on new real-time data to show its effectiveness.

Authors of [15] had used support vector machine (SVM) for predicting protein stability that changes from single amino acid mutations. This inspired us to use SVM in detecting stress from EEG signals.

In [16], authors have proposed a strategy for identifying the stress by using the EEG signals. They had used K-means clustering method for data classification and technique.

Gaurav et al. [17] have portrayed a strategy to distinguish mental stress level dependent on physiological parameters. They had used SVM based on the binary classifier and classified stress in 2 levels and 3 levels, respectively. Out of 41 volunteers, 30 subjects had been correctly classified in the binary classifier and 26 subjects were identified correctly in the ternary classifier.

In [18], authors have proposed mental stress discovery utilizing phonocardiography signals. Precompetitive (or exam related) mental stress was identified from the S1-S1 interim of PCG signals, also termed as Interbeat Interval (IBI). They had also performed another experiment; Kruskal–Wallis statistical test was used for identifying and extracting various features. The extracted features are then fed into SVM based on the least square classifier.

Authors of [19] have analyzed the effect of English and Urdu music tracks on human stress level. The EEG signals were collected while the subjects were listening either to English or Urdu music. Authors had implemented algorithm based on 4 classifiers, namely, sequential minimal optimization, stochastic decent gradient, logistic regression (LR), and multilayer perceptron. They classified stress into 2 and 3 classes. It was concluded that females are more responsive than males to music.

An extensive structure for the initial identification of mental stress by breaking down varieties in electroencephalogram (EEG) and electrocardiogram (ECG) signals has been proposed in [20]. The prediction of treatment efficacy compared to accuracy had been emphasized. The model explained in the paper defined the stress in 4 classes. The results had shown significant difference in the stress and the control conditions.

In another research illustrated in [21], a technique has been proposed to consequently perceive laborers' stress in building locals. Authors had extracted features in frequency and time domain using fixed and sliding window techniques. They had shown that fixed window approach along with the Gaussian support vector machine resulted in highest accuracy. Authors also collected cortisol, stress hormone from workers during working hours, to classify into low and high stress.

In [22], a machine learning-based approach has been proposed to deal with driving-actuated stress. They had used 3 classifiers, namely, SVM, neural networks, and Random Forest, to classify the EEG signals collected from the subjects. The study concluded by stating SVM performed better during rest and stress states, over others.

In [23], two sparsity based techniques are used to remove the eye blink artifacts, namely, Morphological Component Analysis (MCA) and K-SVD. Results of both algorithms were correlated with the developed FORCe method. K-SVD algorithm removes eye blink better than the MCA algorithm.

Qi1 [24] discussed the elimination of the EOG product from EEG signals using Recursive Least Square (RLS) algorithm. In addition to it, Second-order Blind Identification (SBI) algorithm was also used.

In [25], authors employed low-cost wireless EEG headset for quantifying various cognitive stress states on the single-trial basis. Here, Stroop-type color-word interference test was conducted for eliciting the mild stress responses when recording the scalp EEG of 18 subjects. Then, the computational feature was extracted from EEG signals during each stimulus presentation. Then, the feature extraction output was provided as the input to quadratic discriminant analysis, logistic regression, and k-nearest neighbor classifier for classification. Here, the accuracy was found better, but failed to examine cognitive stress in real time.

Authors of [26] have developed a multiclass support vector machine (SVM). To identify and classify stress, authors had added error correction code (ECOC) to SVM. To better record stress from PFC region, a limited number of EEG-electrodes were used. Thus, classification of stress resulted in average accuracy.

The research in [27] presented stage-wise methodology for stress classification in humans. The method suggested in this paper can prepare classification model in limited time and increased the accuracy. Data was collected from volunteers, who wore single channel EEG device.

In [28], a technique was developed using multiclass support vector machine for brain stress classification. Although this framework achieves better performance in monitoring the brain network states, it failed to consider samples for training process.

The research carried out in [29] employed SVM classification for stress classification using EEG signals. However, the accuracy was found better, but failed to examine a large data for stress and nonstress subjects.

In another research carried out in [30], a fNIRS-based brain-computer interface for EEG stress classification was developed. Here, the accuracy was found better, but failed to include the bundle type dataset to achieve minimal error.

## 3. Proposed Methodology

**Figure 1:**
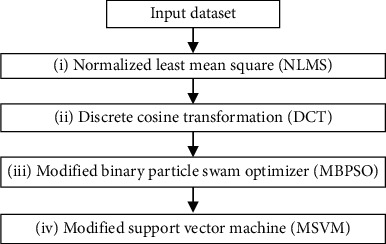
Block diagram of EEG data processing algorithms.

The suggested method has four main phase algorithms, namely, (i) preprocessing, (ii) feature extraction, (iii) feature selection, and (iv) classification (stress level identification), besides data acquisition stage. The block diagram of the suggested methodology is shown in Figure 1.

### 3.1. Data Acquisition

**Figure 2:**
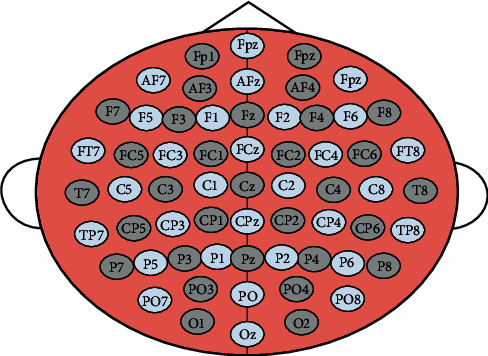
The 32-electrode location map used in the data acquisition step as per 10–20 international system.

The dataset used in the work was taken from [31]. Around 14 human subjects with normal vision (7 women and 7 men) of an average age of 26 years (ranging between 22 and 46) volunteered for the study. The subjects sat in a dimly lit room, at a distance of 110 cm from a colored computer screen having a block of 100 images. Subjects were also holding a touch-sensitive button. The 32 electrodes mounted on the EEG cap recorded the associated EEG. Electrodes were placed in 10–20 system and are divided into 2 categories: frontal electrodes and occipital electrodes. A SynAmps recording system (Neuroscan Inc.) was attached to a PC, which documented data at 1000 Hz. In addition, 500 Hz of a low-pass filter was used and impedance was maintained below 5 kOhms. Images were divided in targets and nontargets (distractors) while shown to subjects. Targets are photographs including images of mammals, reptiles, arthropods, fish, and birds and nontargets include images of outdoor and indoor places, natural landscapes or urban sites, fruit, vegetables, trees, and flowers. There are two types of tasks, namely, categorization or recognition. Categorization had 1000 pictures (50% distractors and 50% objectives), shown only once to subjects. During the categorization task, targets and nontargets had an equal chance in every block of a hundred pictures so the target photograph assigned to the block was viewed fifty times between 50 completely different nontarget images. Fifteen targets (a total of 210 targets) and therefore the same 750 nontarget stimuli were tested by every one of the fourteen subjects. One hundred forty targets (10 pictures per subject) contained an animal within the 210 pictures used as targets and were, therefore, closest to the target images employed in the categorization method. Stimuli onset between 2 images was random from 1800 ms to 2200 ms. Subject's response as go/no-go is recorded. On seeing the target image, they had to release the button as soon as possible while, on watching the nontargets, the button should not be released. Time to react is 1000 ms, beyond which it was recorded as no-go or nontarget. This experiment was performed for 2 days. Subjects were screened for alternating tasks of categorization and recognition in a session of 10 blocks each and corresponding EEG is documented. For the method of recognition task, every test block was preceded by a learning stage. During training or learning phase, the target images were flashed perpetually for twenty ms (comparable to the test circumstances). In order to respond to images accurately and quickly in the respective sequence of images, participants were advised to memorize images. The block of testing began instantly after the learning stage.  Figure 2 shows the international system used for 32-electrode setup for the said experiment.

### 3.2. Preprocessing

The preprocessing techniques remove undesirable artifacts from the EEG signal and remove the eye blinking artifacts [32]. Normalized least mean square (NLMS) technique is used [33] for preprocessing. The NLMS algorithm updates the coefficients of an adaptive filter as tap-weight at (*m* + 1) iterations as per the following:

(1) $$\begin{array}{c} f\left(m+1\right)=\bar{f}\left(m\right)+\mu \cdot e\left(m\right)\frac{\bar{x}\left(m\right)}{{\left\|\bar{f}\left(m\right)\right\|}^{2}}, \end{array}$$

where *μ* is a time varying step size and should be carefully chosen, as large value can lead to algorithm instability while less value can make algorithm large time converge. *e* (*m*) is estimated error and its conjugate is found at *m*^th^ iteration. The modification in NLMS is normalized to squared Euclidean norm of *μ* (*m*) at (*m* + 1) iteration. Based on the above process, the input raw signal is preprocessed and the resultant output was fed to the next step, feature extraction.

### 3.3. Feature Extraction

Feature extraction retrieves the most significant features from the input signal. In the proposed algorithm, features were extracted using the discrete cosine transform (DCT). In the two stages of the DCT, there exist 2 coefficients, each concentrated on the low and high frequency components. To obtain the DCT coefficients, the DCT was applied on the input signal, in the first phase. Later, in the second phase, feature vectors were constructed using some of the selected coefficients. The DCT input a (*n*) is a set of *N* data values (EEG samples, audio samples, or other data) and the output *A* (*n*) is a set of *N* Discrete Cosine Transform coefficients [34].

(2) $$\begin{array}{c} A\left(n\right)=\propto \left(p\right)\sum_{n=0}^{N-1}a\left(n\right)\cos\left[\frac{\pi \left(2n+1\right)p}{2N}\right],\\ a\left(n\right)=\sum_{k=0}^{N-1}\propto \left(p\right)A\left(n\right)\cos\left[\frac{\pi \left(2p+1\right)n}{2N}\right],\\ \propto \left(p\right)=\left\{\begin{array}{cc} \sqrt{\frac{1}{N}}, & p=0,\\ \sqrt{\frac{2}{N}}, & p\neq 0. \end{array}\right. \end{array}$$

The first coefficient *A* (0) is called the *DC* coefficient and rests are referred to as *AC* coefficients. The *DC* coefficient encompasses mean from the input signal. The DCT displays good energy compaction for highly correlated signals. The feature vector has been generated from the input signal by utilizing the DCT. Some of the features extracted were mean, median, standard deviation, kurtosis, skewness, variance, short time energy, waveform length, frequency value, SNR, and mean absolute deviation. There are a total of 22 features extracted.

### 3.4. Feature Selection

In order to choose the optimal feature for stress level identification issue, the modified binary particle swarm optimization (MBPSO) algorithm was utilized. In the proposed method, traditional particle swarm optimization algorithm was modified by means of binary value to modified binary particle swarm optimization (MBPSO) [35]. The new random particle for selecting the new optimal feature set generation is used as follows:

(3) $$\begin{array}{c} v_{p\;d}^{\text{new}}\;=\;wv_{p\;d}^{\text{old}}+\left[c_{1}r_{1}\left(pbest_{p\;d}-x_{p\;d}^{\text{old}}\right)\right]\;+\;\left[c_{2}r_{2}\left(gbest_{p\;d}-x_{p\;d}^{\text{old}}\right)\right], \end{array}$$

where *gbest* and *pbest* are global best and local best values of fitness function from entire iterations and current iteration, respectively, *v*_*p* *d*_^new^ represents the new velocity particles, and *v*_*p* *d*_^old^ represents the previous velocity of particle *p* in *d*th iteration. The *c*_1_ = *c*_2_ = 2 are constant variable. Each particle representing the feature selection is given by

(4) $$\begin{array}{c} S\left(V_{p\;d}^{\text{new}}\right)=\frac{1}{1+e^{-v_{p\;d}^{\text{new}}}}. \end{array}$$

The feature on renewal is calculated by the function *S*(*v*_*p* *d*_^new^), in which the speed value is *V*_*p* *d*_^new^. The *x*_*p* *d*_^new^ represents the new particle in the MBPSO technique. If  *S*(*V*_*p* *d*_^new^) is larger than a randomly formed disorder number within (0, 1), then the feature is selected; otherwise, the feature is not selected. Thus, the final optimal feature set has been extracted by utilizing the MBPSO technique.

### 3.5. Classification (Stress Level Identification)

The proposed stress level identification is finally performed with the help of support vector machine (SVM). Here, the Modified Whale Optimization Algorithm (MWOA) is used to select the optimal kernel in the SVM classifier. The kernel function used in support vector machines (SVM), *K* (*x*_*n*_, *x*_*i*_), transformed the original data space into a new space with a higher dimension. The accuracy of the WOA algorithm as discussed in [36] is enhanced by assigning fitness objective function values to random numbers. This is discussed as (i) encircling prey phase, (ii) exploitation (bubble-net attacking) phase, and (ii) exploration (search for a prey) phase. For hunting, whales use the spiral bubble technique which is depicted in the WOA.

#### 3.5.1. Encircling Prey Phase

During the said phase, the whales, which are search agents looking for the prey, identifies the position of the prey, and then they start circling them. This can be formulated using the following:

(5) $$\begin{array}{c} D=\left|C\cdot X^{*}\left(t\right)-X\left(t\right)\right|, \end{array}$$

(6) $$\begin{array}{c} X\left(t+1\right)=X^{*}\left(t\right)-A.D, \end{array}$$

where *A* and *C* are coefficient vector, the current iteration is recorded by *t*, and *X*^*∗*^ represents the best position vector obtained till current phase while *X* indicates the overall best value of respective position vector. The coefficient vectors, *A* and *C*, are evaluated as follows:

(7) $$\begin{array}{c} A=2a\cdot r_{1}-a,\\ C=2\cdot r_{2}, \end{array}$$

where *r*_1_, *r*_2_ are random numbers that decrease linearly during exploitation and exploration phases.

#### 3.5.2. Exploitation (Bubble-Net Attacking) Phase

**Figure 3:**
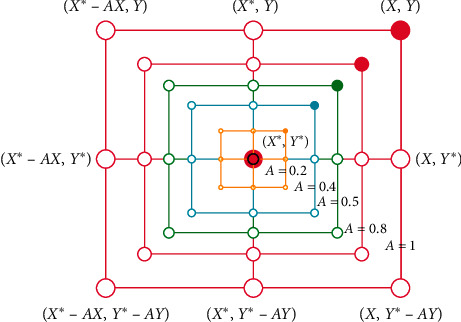
WOA shrinking encircling mechanism [37].

**Figure 4:**
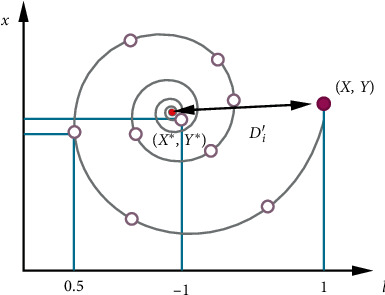
WOA spiral updating position [37].

This phase marks the implementation of two techniques, namely, shrinking encircling technique (Figure 3) and spiral updating position (Figure 4) [36].

Whales update their position as per the following equation:

(8) $$\begin{array}{c} X\left(t+1\right)=De^{bt}\cos\left(2\pi t\right)+X^{*}\left(t\right), \end{array}$$

where *D*=|*X*^*∗*^(*t*) − *X*(*t*)| indicates the distance between the current whale's position and the prey's position and *b* represents a constant which describes the movement of whales in a spiral path. This switching can be expressed mathematically by

(9) $$\begin{array}{c} X\left(t+1\right)=\left\{\begin{array}{cc} X^{*}-A\;D, & p<0.5,\\ De^{bt}\cos\left(2\pi t\right)+X^{*}\left(t\right), & p\geq 0.5, \end{array}\right. \end{array}$$

where *p* is a random number over the interval [0, 1].

#### 3.5.3. Exploration (Search for a Prey) Phase

This phase explores the property of the whales to refresh their position in accordance with the reference whale, which is selected randomly. Thus, the updation to identify the best whale for a global search is presented by equations (10) and (11) [36]:

(10) $$\begin{array}{c} D=\left|CX_{\text{rand}}-X\right|, \end{array}$$

(11) $$\begin{array}{c} X\left(t+1\right)=X_{\text{rand}}-A\;D, \end{array}$$

where *X*_rand_ is a random value that identifies the location of the randomly selected whale from the pool of available whales. The iteration will continue till it reaches the maximum iteration.

Further, the WOA is enhanced to the MWOA by calculating random values, *r*_1_ and *r*_2,_ used in encircling prey phase as per equations (12) and (13) [33–38]:

(12) $$\begin{array}{c} r_{1}=\frac{f_{\max}+f_{\min}}{2}, \end{array}$$

(13) $$\begin{array}{c} r_{2}=\frac{f_{\max}-f_{\min}}{2}, \end{array}$$

where *f*_max_ is maximum value of fitness function and *f*_min_ is minimum value of fitness function from MBPSO.

### 3.6. The Objective Function

The purpose of calculating objective function is to enhance the accuracy of the WOA for the stress level identification and is given by

(14) $$\begin{array}{c} \text{Fit}=\;\underset{i=1:n}{\max}\left(\frac{\left(t_{p}+t_{n}\right)}{\left(t_{p}+t_{n}+f_{p}+f_{n}\right)}\right), \end{array}$$

where *t*_*p*_ and *t*_*n*_ correspondingly represent true positive and true negative and *f*_*p*_ and fn correspondingly represent false positive and false negative.

## 4. Results and Discussion

**Figure 5:**
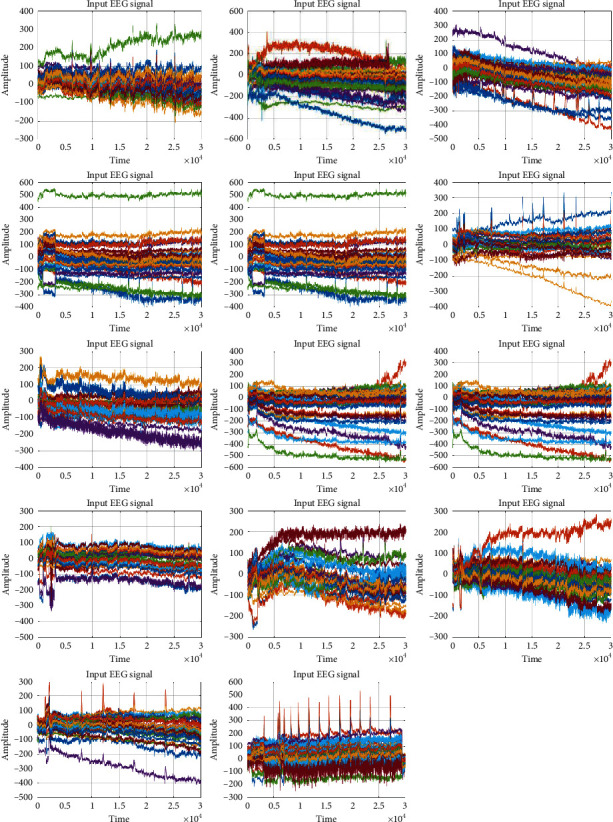
EEG signals of 14 different subjects with 31 attributes of each. These images were generated before preprocessing step.

The proposed optimal stress level predictions have been computed using MATLAB. In this section, the proposed strategy is explained through the results. The input EEG signal dataset is taken from [31]. For each of the 14 subjects (25 files for each subject), preprocessing was performed using the NLMS algorithm. The adaptive filter removes the eye blink artifacts. The input signals have 31 different attributes as shown by different colors in Figure 5. The results of Figure 4 are images before preprocessing step. The subjects are named as *cba*, *clm*, *ega*, *fsa*, *gro*, *hth*, *lmi*, *mba*, *mma*, *mta*, *pla*, *sce*, *sph*, and *wpa*.

**Figure 6:**
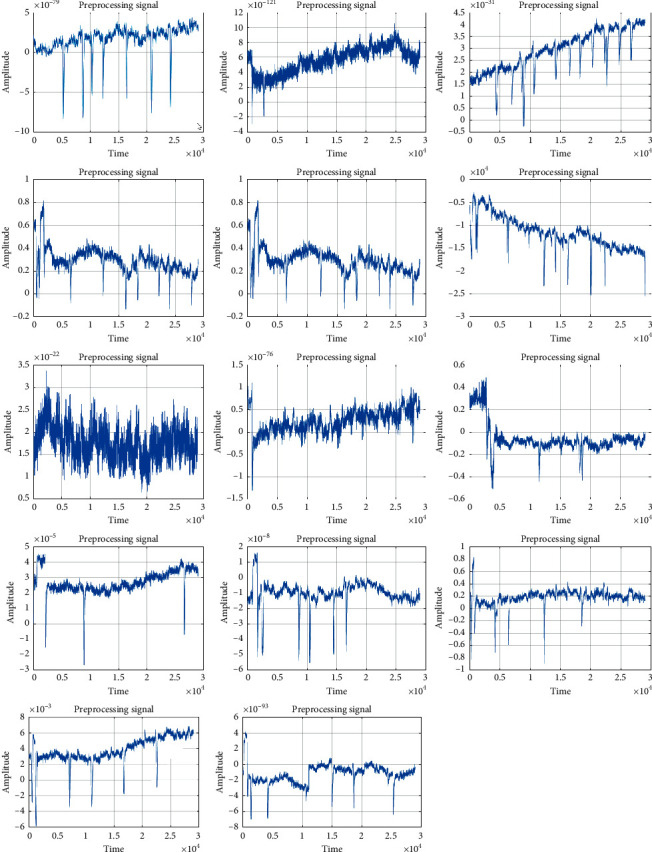
Images generated after applying NLMS on different subject. The images so shown are result of after preprocessing step. It removed the eye blink artifacts and other noisy artifacts.

Further, Figure 6 shows the NLMS output after eye blink removal for 14 subjects. This shows the results after preprocessing step. In the preprocessing, each input signal has 31 different attributes. The NLMS preprocessing technique is used to uniquely identify a signal which depends on 31 attributes of each subjects. Hence, the signal data has been compressed which is represented in the graphical plots.

**Figure 7:**
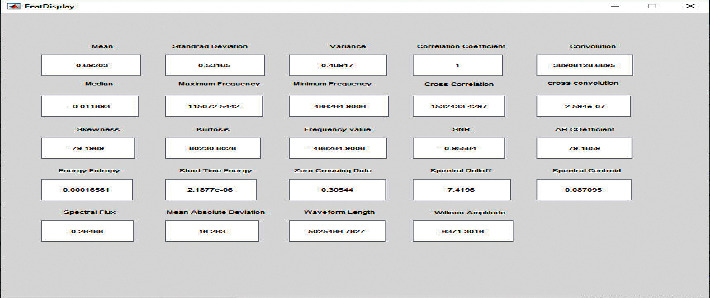
24 features extracted and their respective values using DCT (for *cba* subject).

Figure 7 shows a total of 24 features extracted (for *cba* subject) using the DCT. The DCT is used to compress the signal from different attributes and the feature extraction has been computed for the vector subject. The DCT converts the original signal into vectorized outcomes in which it is used to enhance the classification process to acquire the different long term stress level classification.

**Figure 8:**
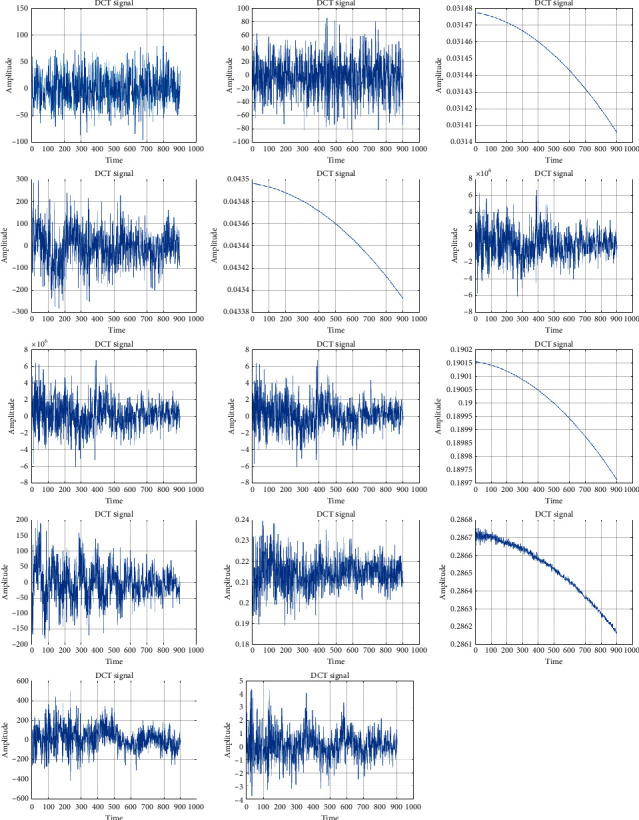
The DCT plot for the signals whose features were extracted in Figure 6.

While Figure 8 shows the DCT output for all 14 subjects. From the DCT signal, the proposed features are extracted and then the optimal features are selected by MBPSO. The algorithm runs for 100 iterations to find the best among the 24 extracted. Finally, the stress level identification is done by SVM. It should be noted that, from 25 files for each subject, 18 were considered for training and 7 were considered for testing purposes.

Among these 24, 10 best features are selected for both current and proposed algorithm, respectively. For one of the subjects, *cba*, selected features are shown in Table 1. Here it should be noted that the algorithm for selecting best features had run for 100 iterations and the best results after each iteration were noted. The iterations which had best result among each other are selected for the next step.

The performance analysis is based on the confusion matrix parameters. The results are assessed by accuracy, sensitivity, specificity, and *F*1 score.

(15) $$\begin{array}{c} \mathrm{Accuracy}\;=\frac{\text{TP}+\text{TN}}{\text{TP}+\text{TN}+\text{FP}+\text{FN}},\\ \mathrm{Sensitivity}\;=\frac{\text{TP}}{\text{TP}+\text{TN}},\\ \mathrm{Specificity}\;=\frac{\text{TN}}{\left(\text{TN}/\text{FP}\right)},\\ F1\text{ score}=\frac{2\text{TP}}{\left(2\text{TP}+\text{FP}+\text{FN}\right)}, \end{array}$$

where TP refers to true positive, TN refers true negative, FP refers to false positive, and FN refers to false negative.

Table 2 demonstrates the overall result attained for all subjects. The performance analysis comprising accuracy, sensitivity, specificity, and *F*1 score has been evaluated. The different techniques were such as MPSO_MWOA_SVM (proposed technique), PSO_MWOA_SVM, MBPSO_WOA_SVM, and MBPSO_SVM. From the comparative analysis, the proposed technique shows the better outcomes when compared with the other techniques.

**Figure 9:**
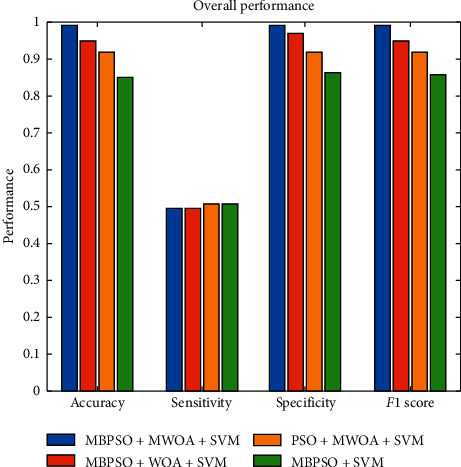
Average performance comparison of various techniques used along with particle swarm optimization (PSO) and modified binary particle swarm optimization (MBPSO).

Figure 9 is graphical representation of Table 2 for easier understanding.

The effectiveness of the proposed strategy (SVM + MWOA) contrasted with current method (SVM + WOA) is presented in Table 3.

**Figure 10:**
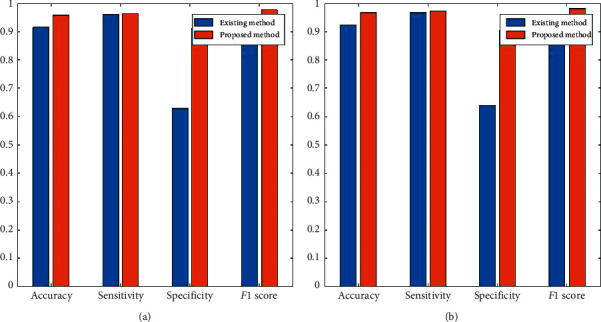
Comparative results of 2 subjects (*cba* and *clm*) based on confusion matrix parameters. The results show the comparison of existing and proposed method on 4 different parameters. It also explains the effectiveness of proposed method over existing. (a) Parameter of *cba*. (b) Parameter of *clm*.

The average computational time taken by the proposed algorithm is 46 sec.  Figure 10 shows the histogram of the results in Table 3. Average accuracy, sensitivity, specificity, and F1 score for existing algorithm are 0.9165, 0.9623, 0.6193, and 0.9523, while for proposed algorithm, average accuracy, sensitivity, specificity, and *F*1 score are 0.9602, 0.9689, 0.9087, and 0.9800.

Stress level was categorized into 4 different classes. On the basis of above parameters, files in each subject were categorized as low, high, medium, or no stress level. The tasks were carried out in 2 days.  Table 4 shows the stress level identification for each task carried out in 2 days, for subject *cba*.

**Figure 11:**
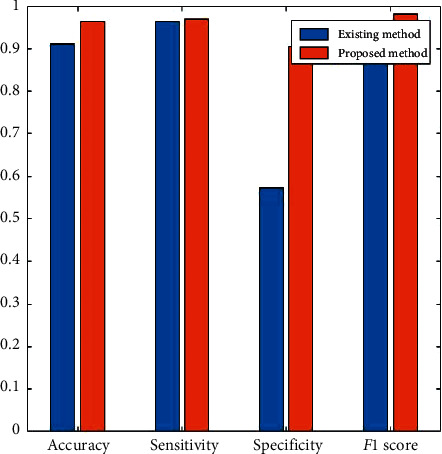
Average performance comparison of the input EEG dataset.

Figure 11 shows the overall average performance of the entire input dataset.

**Figure 12:**
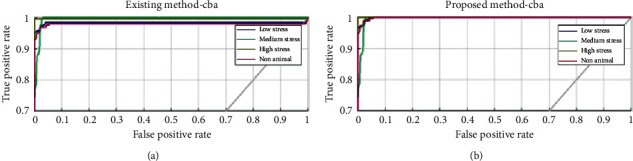
Existing (nonmodified SVM) and proposed (modified SVM) ROC plots for subject *cba*. (a) Exiting method (*cba*). (b) Proposed method (*cba*).

When the various results are analyzed, it is found that the proposed method (SVM + MWOA) attains the maximum performance value compared to the original method (SVM + WOA). The graphical representation of the ROC plot for *cba* is shown below in Figure 12.

From the outcomes, it is observed that the proposed stress level identification accomplishes better classification accuracy when contrasted with current techniques.

## 5. Conclusion

The test outcomes depict the effectiveness of the stress level identification by figuring the right and accurate estimation with the maximum accuracy value. The proposed method accomplishes the maximum accuracy, sensitivity, specificity, and *F*1 score with values 96.36%, 96.84%, 90.8%, and 97.96%. From the analyses of the above results, we found that adding fitness function in the WOA could enhance the efficiency of the algorithm. Also, it can be well concluded after the discussions of the results that the set of algorithms chosen for the study suits best to enhance the efficiency of our proposed algorithm. Eye blink artifacts had been removed during preprocessing step using NLMS. Later, for feature extraction, the DCT had been used and further MBPSO has been for feature selection. Further for classification step, SVM has been used along with the WOA, which was further modified for classifying stress effectively. Although an attempt to classify the stress level for each subject using the WOA has been made in this research, we still feel if few other biological features along with EEG can be added, then we can detect level stress much accurately and efficiently. Besides, implementation of the WOA and its modified version has been successfully done in this application of stress detection. The future work can also be broadened by utilizing some other independent component analysis and other machine learning techniques to improve the characterization exactness.

## Figures and Tables

**Table 1:** Feature selected for the subject *cba*.

Feature selected using SVM + WOA (current algorithm)	Feature selected using SVM + MWOA (proposed algorithm)
Median	Skewness
Frequency value	Mean absolute deviation
Standard deviation	Wilson amplitude
SNR	Spectral centroid
Standard deviation	Zero crossing rate
Waveform length	Zero crossing rate
Spectral flux	Mean
Mean absolute deviation	Mean absolute deviation
Variance	Spectral flux
Skewness	Cross convolution

**Table 2:** Effectiveness of different technique for overall subjects.

Techniques	Accuracy	Sensitivity	Specificity	*F*1 score
MBPSO + MWOA + SVM	0.989637	0.497382	0.989691	0.989583
MBPSO + WOA + SVM	0.948187	0.497268	0.968421	0.947917
PSO + MWOA + SVM	0.917098	0.508475	0.915789	0.918367
MBPSO + SVM	0.852041	0.508982	0.863158	0.854271

**Table 3:** Effectiveness of proposed stress level, identification for 14 subjects in the dataset.

Subjects		Metrics			
		Accuracy	Sensitivity	Specificity	*F*1 score
*Cba*	Existing	0.9226	0.9664	0.6405	0.9558
	Proposed	0.9141	0.9716	0.9074	0.9815
*Clm*	Existing	0.9162	0.9611	0.6272	0.9521
	Proposed	0.9586	0.9632	0.9124	0.9769
*Ega*	Existing	09195	0.9646	0.6116	0.9543
	Proposed	0.9645	0.9692	0.9095	0.9804
*Fsa*	Existing	0.9050	0.9610	0.5920	09449.
	Proposed	0.9660	0.9714	0.9078	0.9813
*Gro*	Existing	0.9180	0.9618	0.6262	0.9532
	Proposed	0.9592	0.9645	0.9055	0.9773
*Hth*	Existing	0.9184	0.9608	0.6363	0.9534
	Proposed	0.9671	0.9722	0.9111	0.9812
*Lmi*	Existing	0.9213	0.9636	0.6345	0.9552
	Proposed	0.9623	0.9675	0.9122	0.9790
*Mba*	Existing	0.9127	0.9626	0.5969	0.9501
	Proposed	0.9660	0.9707	0.9112	0.9813
*Mma*	Existing	0.9142	0.9586	0.6262	0.9509
	Proposed	0.9627	0.9699	0.9052	0.9794
*Mta*	Existing	0.9141	0.9608	0.5986	0.9512
	Proposed	0.9674	0.9729	0.9038	0.9821
*Pla*	Existing	0.9184	0.9596	0.6363	0.9536
	Proposed	0.9649	0.9705	0.9055	0.9806
*Sce*	Existing	0.9173	0.9611	0.6307	0.9527
	Proposed	0.9614	0.9664	0.9060	0.9787
*Sph*	Existing	0.9262	0.9681	0.6271	0.9583
	Proposed	0.9642	0.9650	0.9154	0.9806
*Wpa*	Existing	0.9072	0.9622	0.5862	0.9465
	Proposed	0.9646	0.970	0.9101	0.9803

**Table 4:** The subject *cba* files (different tasks) divided into various stress levels (low, medium, high, and no stress).

Day 1		Day 2	
Subject *cba*	Stress level	Subject *cba*	Stress level
*Cba*d11	Low	*Cba*d21	Low
*Cba*d110	Low	*Cba*d210	Low
*Cba*d111	Medium	*Cba*d211	No stress
*Cba*d112	High	*Cba*d212	Low
*Cba*d113	Low	*Cba*d22	No stress
*Cba*d12	High	*Cba*d23	High
*Cba*d13	Medium	*Cba*d24	Low
*Cba*d14	Low	*Cba*d25	Medium
*Cba*d15	No stress	*Cba*d26	No stress
*Cba*d16	High	*Cba*d27	Low
*Cba*d17	Low	*Cba*d28	High
*Cba*d18	Medium	*Cba*d29	Medium
*Cba*d19	No stress		

## Data Availability

Data used to support the findings of this study were obtained from this site: https://sccn.ucsd.edu/∼arno/fam2data/publicly_available_EEG_data.html.
